# Direct oral anticoagulation versus no therapy or antiplatelet for stroke prevention in patients with atrial fibrillation and history of intracranial hemorrhage: a systematic review and meta-analysis

**DOI:** 10.3389/fmed.2025.1570809

**Published:** 2025-04-25

**Authors:** Denis Sablot, Nicolas Gaillard, Faouzi Belahsen, Sara Rivas Lamelo, Adrian Dumitrana, Carole Plantard, Mohamed Aziz Daghmouri, Mohamed Ali Chaouch

**Affiliations:** ^1^Neurology Department of Perpignan Hospital, Perpignan, France; ^2^Commission of Clinical Research and Innovation, Perpignan, France; ^3^Regional Health Agency of Occitanie, Montpellier, France; ^4^Clinique Beausoleil, Montpellier, France; ^5^Neurology Department, University Hospital Hassan II, University of Fes, Fes, Morocco; ^6^Department of Intensive Care, Montreuil Hospital, Montreuil, France; ^7^Department of Visceral and Digestive Surgery, Monastir University Hospital, University of Monastir, Monastir, Tunisia

**Keywords:** atrial fibrillation, intracranial hemorrhage, direct oral anticoagulants, stroke prevention, meta-analysis, antiplatelet therapy

## Abstract

**Background:**

Patients with atrial fibrillation and a history of intracranial hemorrhage (ICH) face a dilemma when resuming anticoagulation therapy due to the risk of ICH recurrence versus the need for Ischemic stroke (IS) prevention. This study aims to evaluate the safety and efficacy of direct oral anticoagulants (DOAC) compared to no therapy or antiplatelets in these patients.

**Methods:**

We conducted a systematic review and meta-analysis following PRISMA 2020 guidelines. Electronic searches were performed in multiple databases (Cochrane, PubMed, Web of Science, Embase, Google Scholar, Scopus) up to March 1, 2024. We included randomized controlled trials (RCTs) and controlled clinical trials (CCTs) involving patients with atrial fibrillation and prior ICH. Studies compared the group with no therapy or antiplatelets (no-DOAC group). Outcomes assessed included mortality, IS, ICH recurrence, and major bleeding events.

**Results:**

Fifteen studies (8,318 patients) met the inclusion criteria, including 2,226 patients in the DOAC group and 5,936 in the no-OAC group. The major cardiovascular ischemic event was significantly lower in the DOAC group [OR = 0.11; CI 95% (0.03, 0.45); *p* = 0.002]. Ischemic stroke was lower in the DOAC group [OR = 0.53, 95% CI (0.39–0.72), *p* < 0.001]. There was no difference in ICH recurrence [OR = 1.25, 95% CI (0.28–5.71), *p* = 0.77] or major bleeding [OR = 0.63, 95% CI (0.23–1.72), *p* = 0.36]. Mortality rates were similar between groups [OR = 0.75, 95% CI (0.50–1.11), *p* = 0.15], while Heterogeneity was low for most outcomes.

**Conclusion:**

DOACs appear to reduce the risk of IS without increasing mortality or major bleeding in patients with atrial fibrillation and prior ICH. However, the risk of ICH recurrence remains uncertain. These findings suggest a potential role for DOACs in this high-risk population, but further RCTs are needed to confirm these results.

**Systematic review registration:**

Identifier CRD42024587511.

## Introduction

Survivors of intracranial hemorrhage (ICH) with atrial fibrillation (AF) face the highest risk of major adverse cardiovascular events and notably ischemic stroke (IS), with their risk appearing to exceed that predicted by the CHA2DS2-VASc score for individuals with AF who have not experienced a prior ICH (1, 2). As a result, preventing recurrent IS in patients with AF and a history of ICH is a key priority in cerebrovascular care. Closing the auricle can be a favored alternative for these patients (3–5), but in current practice, this is not retained or contraindicated in many cases, and the choice is then limited between resuming anticoagulant treatment, an antiplatelet agent, or therapeutic abstention. Therefore, oral anticoagulation decreases the risk of IS in individuals with AF by nearly two-thirds compared to controls, despite an increased risk of bleeding, while antiplatelet therapy offers a lesser degree of risk reduction (6, 7). However, individuals with a history of intracranial hemorrhage were excluded from the trials that demonstrated these benefits. In addition, adjusted-dose warfarin is effective in preventing strokes in these patients. Its use is constrained by its narrow therapeutic range, interactions with food and other medications, the need for lifelong coagulation monitoring, and mainly, a high risk of both intracranial and systemic bleeding (7). Compared with warfarin, direct oral anticoagulants (DOACs) may be linked to a reduction in stroke or systemic embolic events (8) but, above all, demonstrated a decreased risk of intracranial hemorrhage (6–11). Evaluating the risk–benefit profile of DOACs is essential for patients with AF and a history of intracranial hemorrhage (ICH), as these individuals face a heightened risk of both recurrent stroke and bleeding complications from anticoagulation therapy, especially ICH.

So, this study aims to evaluate the safety and efficacy of DOAC compared to no therapy or antiplatelets in these patients.

## Methods

We conducted this meta-analysis following the PRISMA 2020 (Preferred Reporting Items for Systematic Review and Meta-analysis) guidelines (12). To evaluate its quality, we employed the AMSTAR 2 (A Measurement Tool to Assess systematic Reviews) tool (13). The study protocol was duly registered in PROSPERO under the identification number CRD42024587511.

### Electronic database searches

We performed a bibliographic electronic literature search and trial registries without language restrictions on March 1st, 2024. The search included multiple databases: the Cochrane Library, PubMed, Web of Science, Embase, Google Scholar, and Scopus. The search strategy included the following keywords: “Randomized Controlled Trials” AND “clinical controlled trials” AND “intracranial hemorrhage” AND “oral anticoagulation” AND “atrial fibrillation.” To identify relevant clinical trials, we manually reviewed the retrieved articles’ reference lists to find additional trials. The research strategies in the different databases are listed in Supplementary File 1.

### Eligibility criteria

#### Studies

We included only RCTs and CCTs, including assigned patients with AF and spontaneous intracranial hemorrhage to long-term use or not of an oral anticoagulant to prevent cardiovascular events. If a subgroup of eligible patients was reported, we retained them for analysis. We excluded review articles, clinical control trials, non-comparative studies, letters to editors, editorials, and case series.

#### Population

The study focused on adults of any gender with AF and a history of spontaneous intracranial hemorrhage (ie, intraventricular hemorrhage, intracerebral hemorrhage, non-aneurysmal subarachnoid hemorrhage).

#### Intervention group

The use of oral anticoagulants was the intervention group (OAC group).

#### Control group

The non-use of oral anticoagulants, or use of antiplatelet or placebo was the control group (no-OAC group).

#### Outcomes

The different outcomes assessed in our study were mortality, cardiovascular mortality, ischemic complications, and bleeding. Cardiovascular mortality was considered if it occurred within 30 days after the onset of a cardiovascular event. We considered the definition of major bleeding proposed by the International Society on Thrombosis and Haemostasis, which includes fatal bleeding, symptomatic bleeding in a critical area or organ (such as intracranial bleeding), bleeding resulting in a hemoglobin drop of ≥2 g/dL, or requiring transfusion of ≥2 units of blood.

#### Study selection

Following independent literature searches conducted by two authors, all abstracts were independently reviewed. The inclusion criteria considered RCTs and CCTs. The full texts of studies that met these criteria were recovered and any disagreements were resolved with a third author. We included ongoing RCTs if they shared the results of a group or subgroup of included patients.

#### Evaluation of study quality and risk of bias

Two authors independently evaluated the selected studies. For the different retained RCTs, we used the Statement of Revision of Consolidated Standards of Reporting Trials (CONSORT) (14). Studies scoring below 13 out of 25 were excluded due to fair quality. The risk of bias was assessed using the Cochrane tool for bias assessment (RoB2) (15). For the retained CCTs, we used the Newcastle-Ottawa scale (NOS) (16) and the Methodological Index For Non-Randomized Studies (MINORS) scale (17).

#### Missing data

The authors were contacted by email in the case of:

- Unclear bias domains or missing primary outcome.
- Specific data extraction or additional analysis is required to obtain an outcome.

Information was extracted from the figures if the data were not numerically reported.

#### Handling continuous data

Continuous data were analyzed using the Cochrane Collaboration 5.3.5 statistical package Review Manager for meta-analysis (18). When the mean and standard deviation (SD) were not provided, these were estimated from the interquartile range (IQR) and median, following the formula described by Hozo et al. (19).

#### Assessment of heterogeneity

To assess heterogeneity, three strategies were used:

1. The Cochrane Chi^2^ test (Q test), Tau2, is the variance of true effects (20).
2. Graphical exploration with funnel plots (21).
3. Sensitivity analysis with a subgroup analysis when applicable.

#### Summary of findings

Two authors independently evaluated the certainty of evidence using The Grading of Recommendations Assessment, Development, and Evaluation (GRADE) (22). Factors considered included study limitations, constancy of effect, imprecision, indirectness, and publication bias. Certainty of evidence was classified as high, moderate, low, or very low. Criteria to improve certainty included a large effect, dose–response gradient, and plausible confounding effect. The Cochrane Handbook for Systematic Reviews of Interventions (Sections 8.5 and 8.7, and Chapters 11 and 12) and GRADEpro GDT software were used to prepare a summary of Findings’ tables, providing explanations for downgrading or upgrading certainty using footnotes with comments.

#### Evaluation of effect size

Meta-analysis was performed using the RevMan 5.3.5 statistical package from the Cochrane Collaboration (23). The standard mean difference (SMD) was selected as the effective measure for continuous data, while odds ratios (OR) with 95% confidence intervals (95% CI) were calculated for dichotomous variables. A random-effects model was applied, with significance set at 0.05.

## Results

### Literature search results

**Figure 1:**
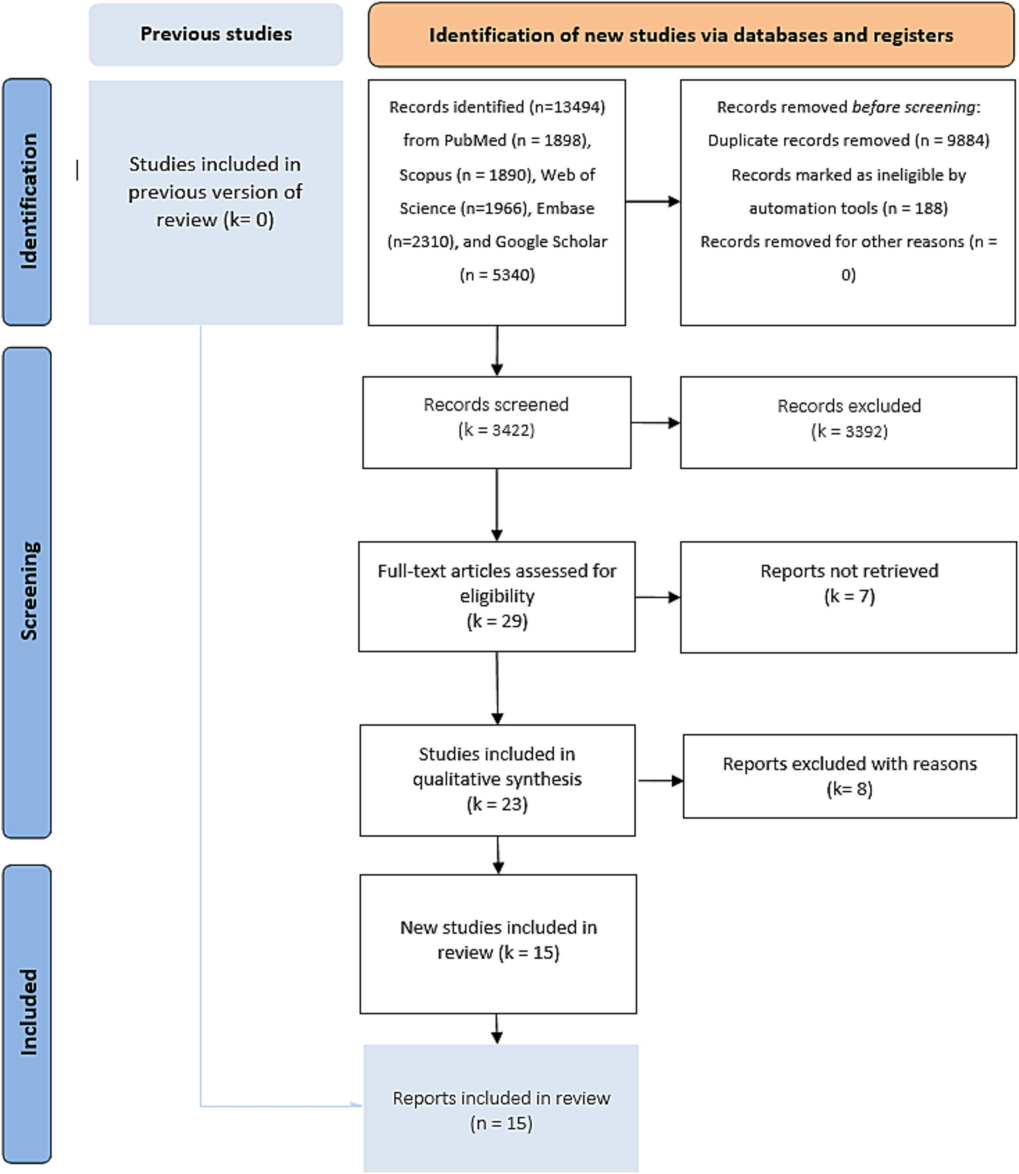
PRISMA Flow diagram of bibliographic research.

After bibliographic research, we screened 13,483 publications. We found 23 potentially eligible studies (Figure 1). We retained 15 studies after assessing the different available full texts. Seven studies were excluded for the following reasons: one systematic review and meta-analysis (24), three studies because the control group was the percutaneous left atrial appendage (3–5), and three studies because the control group was the use of vitamin K antagonists (8–11). Three RCTs were eligible, but the recruitment of patients was ongoing (ASPIRE “NCT03907046” with an expected completion in April 2027, ENRICH-AF “NCT03950076” with an anticipated completion in November 2026, and STROKECLOSE “NCT02830152” with a scheduled study completion in May 2030). Two studies completed patient recruitment, and the follow-up is ongoing (STATICH “NCT03186729” with an estimated primary completion date of December 2026 and A3ICH “NCT03243175” with a primary completion date of June 2023, but no results have been posted yet, and the full study is expected to conclude in 2031). Of the 15 studies included in this systematic review and meta-analysis, two were published RCTs (25, 26), two were RCTs with shared available data in the COCROACH prospective individual participant data meta-analysis of RCTs (26), and 11 were CCTs (27–37). The list of the retained studies and the demographic data of these studies were reported in Tables 1, 2, respectively. The retained studies were published between 2015 and 2023 and conducted between 1995 and 2022. These studies included 8,168 patients: 2226 in the OAC group and 5,936 in the no-OAC group. The mean age ranged from 67.9 to 81.7 years in the OAC group and from 69.1 to 83.6 years in the no-OAC group.

**Table 1:** List of the included studies.

Study number	First author	Journal	Year of publication	Country of origin	Study period	Type of the study	Intervention group	Control group	Outcomes measured	Timing of anticoagulant presumption	Follow-up (months)	MINORS/CONSORT
1.	Schreuder (APACHE-AF)	Lancet Neurology	2021	Netherlands	August 1, 2018, and December 31, 2021	RCT	Apixaban	No anticoagulant therapy	Mortality, major bleeding events, functional outcomes (modified Rankin Scale), quality of life, cardiovascular events	Median 14 days	> 12	20
2.	Salman (SoSTART)	Lancet neurology	2021	Scotland, England, Wales, and Northern Ireland	May 4, 2015, and March 31, 2020	RCT	OAC therapy	No oral anticoagulant	Ischemic stroke, systemic embolism, mortality, major bleeding, functional outcomes (modified Rankin Scale), and quality of life	Median 2 weeks	> 6	25
3.	NASPAF-ICH	–	–	Canada	December 15, 2017, and March 31, 2022	RCT	OAC (apixaban, rivaroxaban, edoxaban, or dagibatran)	Antipalet	Ischemic stroke, ICH, fatal stroke, myocardial infarction, systemic thromboembolism, death, mrs, symptomatic bleed into a critical organ	-	> 0.5	17
4.	ELDERCARE-AF	–	–	Japan	August 5, 2016, and December 27, 2019	RCT	Edoxaban	Placebo	Stroke, systemic embolism, major bleeding, cardiovascular death, minor bleeding, all bleeding		> 6	18
5.	Abrantes	Neurological Sciences	2021	Portugal	January 1, 2009, to May 8, 2018	Single-center retrospective observational study	OAC therapy	No OAC therapy	Mortality, ischemic stroke (IS), and systemic embolism (SE) at both 6-month and 1-year follow-ups.	–	24	20
6.	Komen	European Heart Journal - Cardiovascular Pharmacotherapy	2021	Sweden	July 2011 to June 2018.	Retrospective study with propensity score-matched analyses	NOACs (non-vitamin K oral anticoagulants)	Antiplatelet therapy and no treatment	Functional outcomes at discharge using the modified Rankin Scale (mrs) scores, new-onset ICH, symptomatic hematoma expansion, and gastrointestinal bleeding	–	–	20
7.	Lin	Journal of the American Heart Association	2022	Taiwan	January 1, 2011, to December 31, 2017	Nationwide retrospective cohort study	Oral anticoagulant (OAC) users	Antiplatelet agent users, and non-antithrombotic (non-AT) users	Ischemic stroke and intracranial hemorrhage (ICH). Subtypes of ICH	90 days	–	18
8.	Nielsen	Circulation	2015	Denmark	January 1, 1997, to December 31, 2013	Nationwide cohort study	oral anticoagulant (OAC)	Not receiving any antithrombotic treatment	Recurrent symptomatic spontaneous intracranial haemorrhage, which is a significant major bleeding Symptomatic major vascular events (such as ischaemic stroke, myocardial infarction, and sudden cardiac death), individual symptomatic vascular events, and various types of fatal events. Quality of life using the modified Rankin Scale and EQ-5D-5L questionnaires.	90 days	12	19
9.	Park	Heart Rhythm	2016	Korea	January 1, 2009, to December 31, 2013.	Retrospective observational study	oral anticoagulation therapy (OAT)	Did not take oral anticoagulation therapy (OAT)	Nonfatal stroke (including ischaemic stroke, intracerebral haemorrhage, or subarachnoid haemorrhage) or vascular death various types of haemorrhages (intracerebral, subarachnoid, major extracranial), ischaemic stroke, myocardial infarction, pulmonary embolism, systemic embolism, and all-cause death. Functional outcomes using the modified rankin scale (mrs). Serious adverse events and treatment adherence	–	39.5	20
10.	Perrault	Journal of Stroke	2019	Canada	1995–2015	Monocentric retrospective trial	No treatment	OAC exposure	Ischemic stroke/systemic embolism, recurrent intrcranial hemorrhage, major extracranial bleeding, and all cause of mortality	–	12	18
11.	Poli	Thromb Haemost	2017	Italy	2002 to 2014	Multicenter observational study	oral anticoagulant (OAC) treatment	No antithrombotics	Neurological severity using the National Institutes of Health Stroke Scale (NIHSS). Functional outcomes using the modified Rankin Scale (mrs). The presence of thromboembolic or hemorrhagic events during hospitalization was analyzed. Hematoma volume was with non-contrast computed tomography (CT) on admission. Length of hospital stay and in-hospital mortality.	–	18	20
12.	Sadighi	Eneurologicalsci	2020	United States	2010 to 2017	Observational cohort study	oral anticoagulation (OAC) therapy	Patients who did not resume oral anticoagulation (OAC)	Recurrent ICH, ischemic stroke or systemic emboli, and death.	–	26	20
13.	Sakamoto	Circulation Journal	2019	Japan	September 2014 to March 2017	Retrospective analysis	anticoagulant therapy	Did not receive anticoagulant therapy	Ischemic stroke/systemic embolism (SE) and all-cause mortality. Ischemic stroke/SE, all-cause mortality, and major recurrent bleeding.	–	-	18
14.	Suda	Journal of the neurological science	2023	Japan	February 2017 to January 2020	Sub-analysis of the PASTA registry, which is an observational, multicenter registry	oral anticoagulants (OAC)	The contexts provided do not specify a control group in the study	All-cause mortality within 90 days following an ischaemic stroke, intracranial haemorrhage, or severe gastrointestinal bleed (GIB) in patients with atrial fibrillation.	Median 7 days	–	17
15.	Wu	Medicine	2021	Taiwan	2001 to 2013	Retrospective cohort study	oral anticoagulants (OACs)	No discontinued oral anticoagulants (oacs)	Stroke or cardiovascular death Major adverse cardiovascular events haemorrhagic major adverse cardiovascular events, death from any cause, and death or dependence after one year.	6–8 weeks	12	19

**Table 2:** Demographic data of the retained studies.

Study number	First author	Sample size	OAC group	Non-OAC group	Mean age (OAC/no-OAC) (years)	Sex ratio (M/F)	CHA_2_DS_2_-VAS score (median)	HAS-BLED (median)
1.	Abrantes	95	40	55	74.3 ± 8.9/75.8 ± 10.6	1.2/0.96	3.5 ± 7/4 ± 8	4 ± 4/4 ± 6
2.	ELDERCARE-AF	80	41	39	–	–	–	–
3.	Komen	2,357	311	2046	79.25 ± 9.35/82.6 ± 54	0.91/0.81	4.33 ± 1.71/4.29 ± 72	2.35 (0.91)/2.48 ± 18
4.	Lin	1,566	283	1,283	76.50 ± 21.09/76.20 ± 11.99	0.59/0.58	5.24 ± 3.64/5.23 ± 1.76	–
5.	NASPAF-ICH	30	21	9	78.2 (74–84.7)/78.7 (73.4–83.6)	1.5/1	4/4	–
6.	Nielsen	1752	330	1,035	–	–	–	–
7.	Park	428	254	174	67.9 ± 11.1 /69.1 ± 10.8	0.37/0.28	3.35 ± 1.7/3.16 ± 1.73	3.43 ± 1.13/3.52 ± 1.25
8.	Perrault	483	260	423	81.7 ± 5.8/83.6 ± 5.8	0.85/0.89	3.9 ± 1.3/3.9 ± 1.1	2.6 ± 1.1/2.6 ± 1.1
9.	Poli	393	162	231	77.1/80.3	0.61/0.39	4/4	3/3
10.	Sadighi	98	38	55	74.3 ± 10.5/77.2 ± 10.1	0.65/0.46	–	–
11.	Sakamot	236	41	195	72 (65–80)/68 (59–78)	0.68/0.67	–	–
12.	Salman (SoSTART)	203	101	102	79 (74–85)/79 (74–84)	1.58/1.75	4/4	2/2
13.	Schreuder (APACHE-AF)	101	50	51	77 (74–83)/79 (72–83)	1.17/1.21	4/4	–
14.	Suda	160	108	52	76 (70–81) /77 (71–84)	0.67/0.72	4 (3–5)/4 (3–5)	3 (2–3)/3 (2–4)
15.	Wu	336	186	186	71.0 ± 9.7/71.6 ± 9.5	0.67/0.78	4.1 ± 1.5/4.2 ± 1.5	2.4 ± 0.9/2.5 ± 1.0

### Ischemic stroke

**Figure 2:**
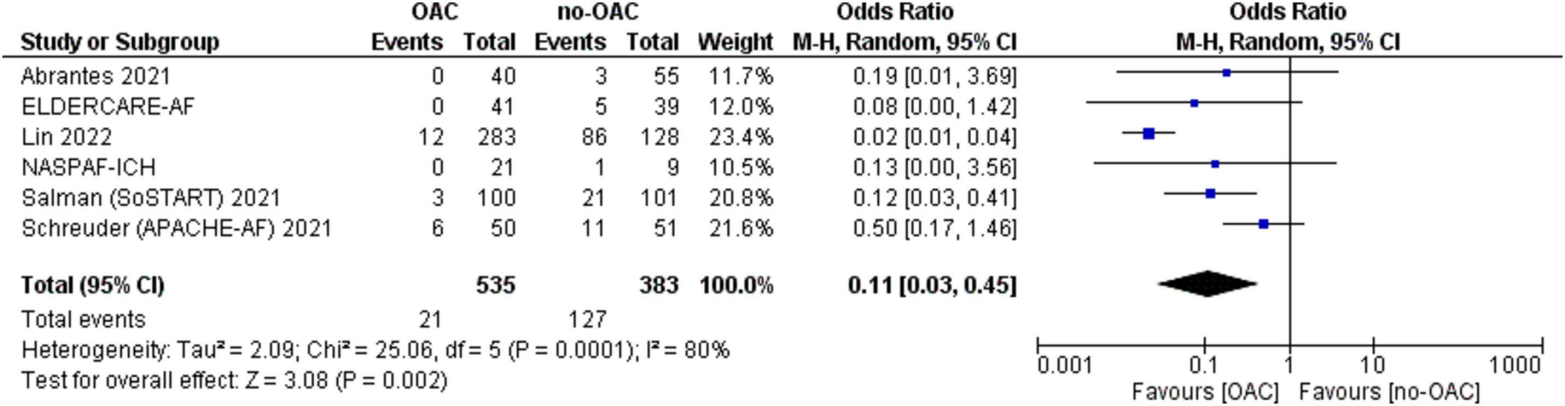
Forest plot of the ischemic stroke.

Ischemic stroke was reported in 11 included studies (Figure 2). It was counted in 66 out of 1835 patients in the OAC group and 232 out of 3,156 patients in the no-OAC group. There was a lower rate of ischemic stroke in the OAC group [OR = 0.53; CI 95% (0.39, 0.72); *p* < 0.001]. In the subgroup analysis, there was no difference in the RCT subgroup [OR = 0.54; CI 95% (0.18, 1.62); *p* = 0.72] and statistically lower ischemic stroke rate in the CCT subgroup [OR = 0.52; CI 95% (0.38, 0.72); *p* < 0.001]. There was no heterogeneity among the different studies.

### Major cardiovascular ischemic events

**Figure 3:**
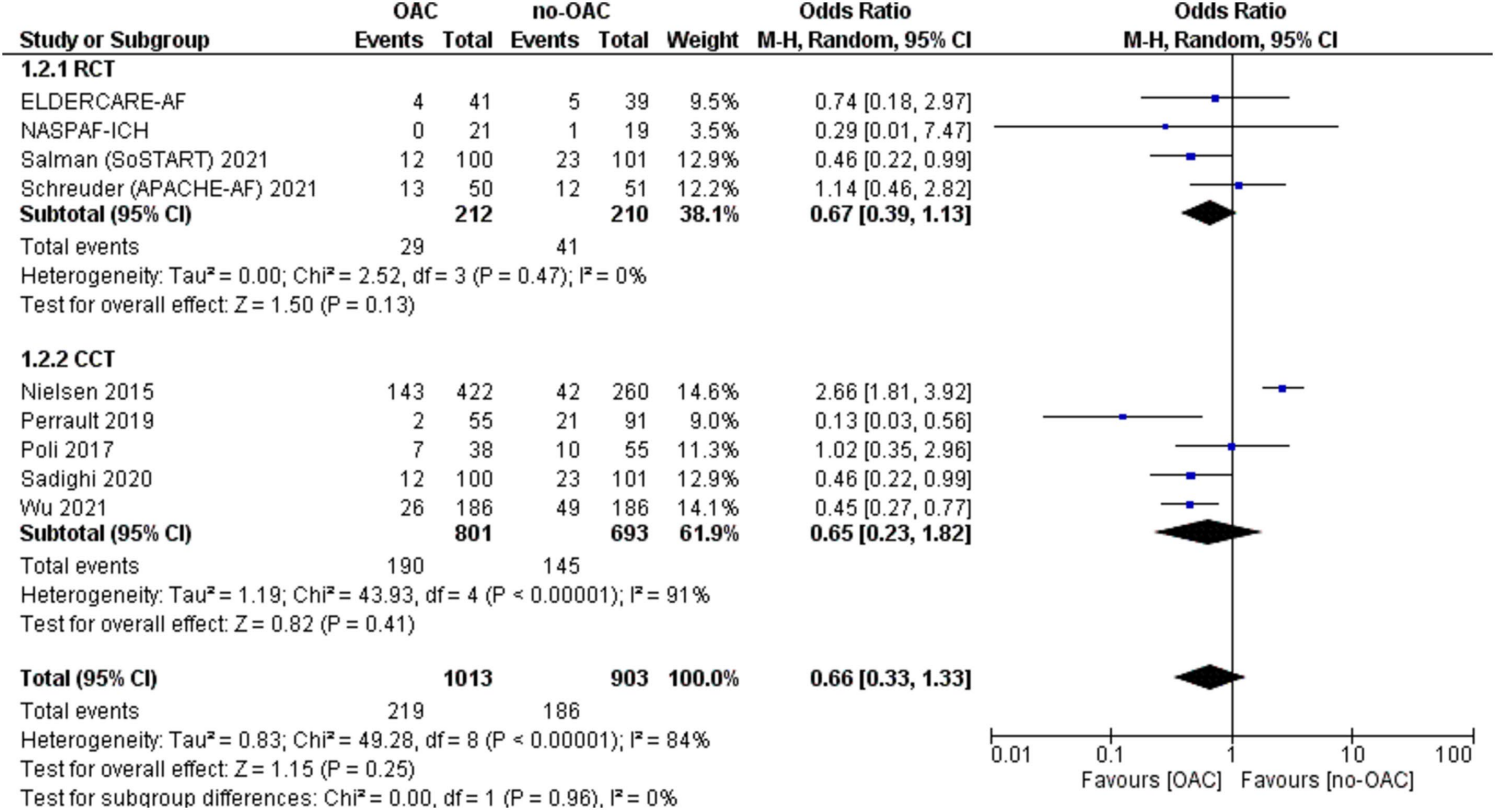
Forest plot of the major cardiovascular ischemic events.

A major cardiovascular ischemic event was reported in six of the included studies (Figure 3). It was counted in 21 out of 535 patients in the OAC group and 127 out of 383 patients in the no-OAC group. There was a lower major cardiovascular ischemic event in the OAC group [OR = 0.11; CI 95% (0.03, 0.45); *p* = 0.002]. There was a low heterogeneity among the different studies (Tau^2^ = 2.53).

### Mortality

**Figure 4:**
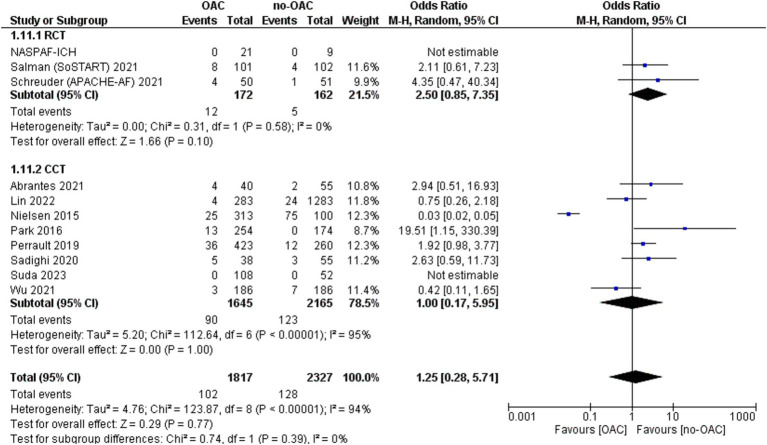
Forest plot of mortality.

The mortality rate was reported in 13 studies (Figure 4). It was counted 388 out of 2,127 patients in the OAC group and 1,166 out of 8,188 in the no-OAC group. There was no difference between the two groups in terms of mortality [OR = 0.75; CI 95% (0.50, 1.11); *p* = 0.15], even in the subgroup analysis of RCT (*p* = 0.74) and CCT (*p* = 0.08). There was a low heterogeneity among the different studies (Tau^2^ = 0.37). There was no difference according to the location of ICH in terms of mortality.

### Major bleeding

**Figure 5:**
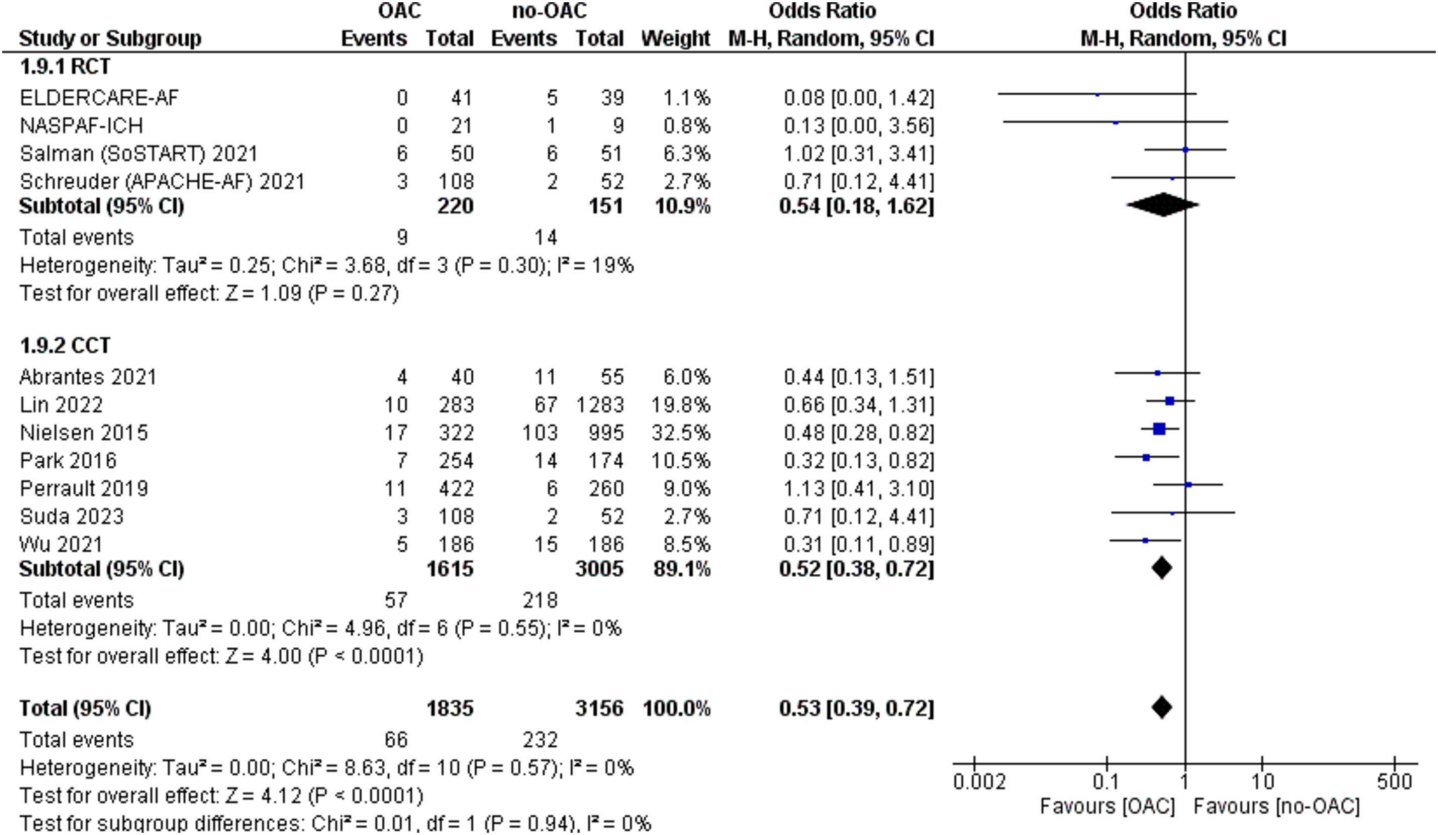
Forest plot of major bleeding.

The major bleeding was reported in 13 included studies (Figure 5). It was counted in 115 out of 1927 patients in the OAC group and 190 out of 2,369 patients in the no-OAC group. There was no difference between the two groups in terms of major bleeding [OR = 0.63; CI 95% (0.23, 1.72); *p* = 0.36], even in the subgroup analysis of RCT (*p* = 0.0.51) and CCT (*p* = 0.33). There was a low heterogeneity among the different studies (Tau^2^ = 2.53).

### Any stroke/SE or cardiac death

**Figure 6:**
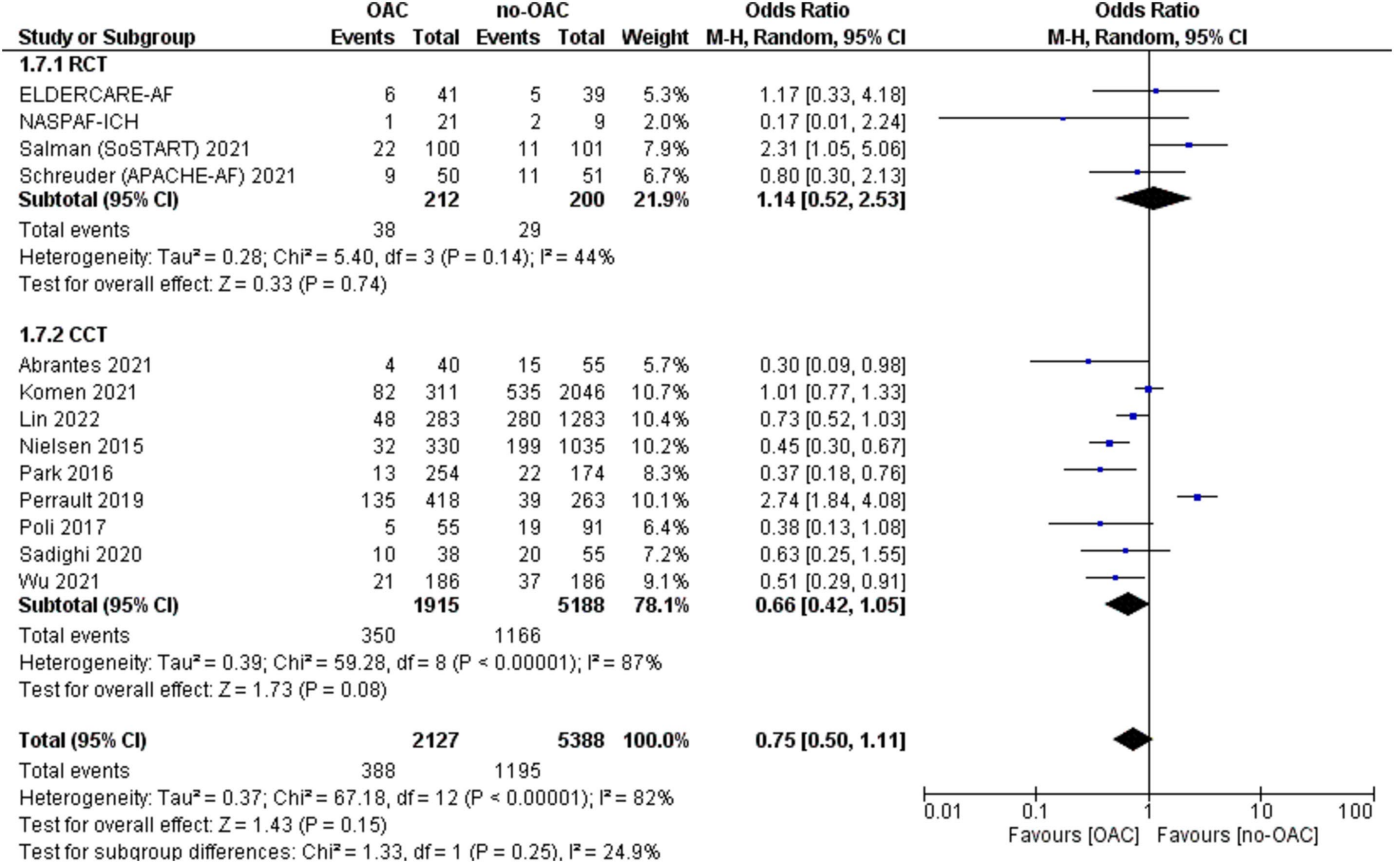
Forest plot of any stroke death.

This outcome was reported in nine studies (Figure 6). It was counted in 219 out of 1,013 patients in the OAC group and 186 out of 903 patients in the no-OAC group. There was no difference between the two groups [OR = 0.66; CI 95% (0.33, 1.33); *p* = 0.25], even in the subgroup analysis of RCT (*p* = 0.13) and CCT (*p* = 0.41). There was a low heterogeneity among the different studies (Tau^2^ = 0.83).

### ICH recurrence

**Figure 7:**
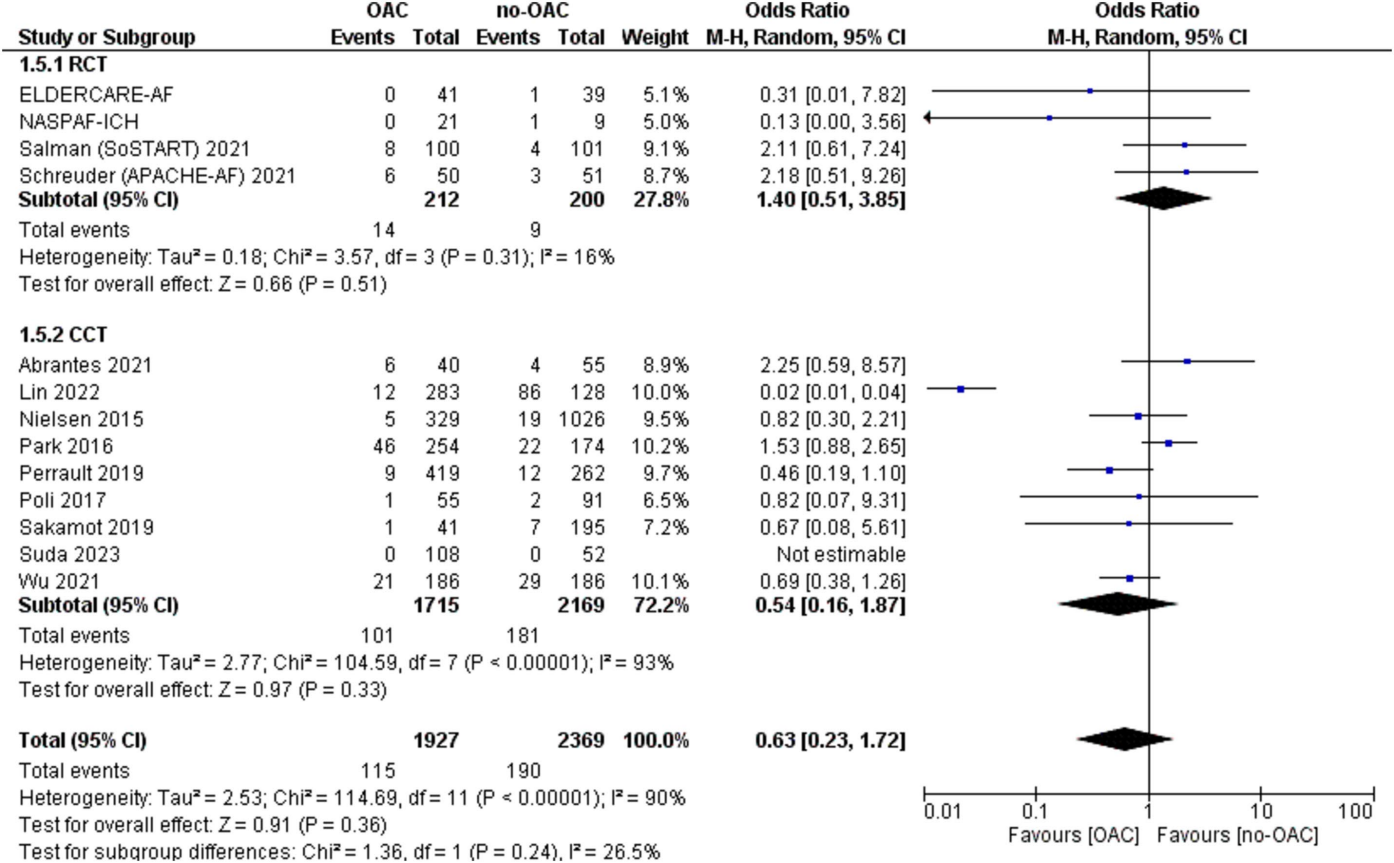
Forest plot of intracranial hemorrhage recurrence.

The ICH recurrence was reported in 11 included studies (Figure 7). It was counted in 102 out of 1817 patients in the OAC group and 128 out of 2,327 patients in the no-OAC group. There was no difference between the two groups in terms of ICH recurrence [OR = 1.25; CI 95% (0.28, 5.71); *p* = 0.77] even in the subgroup analysis of RCT (*p* = 0.10) and CCT (*p* = 1.00). There was a low heterogeneity among the different studies (Tau^2^ = 4.76). There was no difference in the location of ICH in terms of mortality.

### Quality assessment of the included studies and reporting on the effects of OAC use

The quality assessment and risk of bias are presented in Table 1. The risk of bias assessment is presented in Table 3. A Summary of the evidence is presented in Table 4. This review shows that when OAC was used after ICH in patients with AF:

- It reduces major cardiovascular ischemic events
- It may be associated with a lower ischemic stroke rate.
- We do not know if it leads to additional mortality, major bleeding, ICH recurrence, or any stroke death because the evidence is very uncertain.

**Table 3:** Risk of bias assessment.

Randomized controlled trials – RoB 2 tool					
First author	Randomization process	Deviations from intended interventions	Missing outcome data	Measurement of the outcome	Selection of the reported result
Schreuder (APACHE-AF)	Low	High	Low	Low	Low
Salman (SoSTART)	Low	High	Low	Low	Low
NASPAF-ICH	Low	High	Low	Low	Low
ELDERCARE-AF	Low	High	Low	Some concern	Low

Clinical controlled trials – New Castle Ottawa Scale			
First author	Selection	Comparability	Otcomes
Abrantes	****	*	***
Komen	****	**	***
Lin	****	*	***
Nielsen	****	*	***
Park	***	**	***
Perrault	***	**	***
Poli	****	**	***
Sadighi	****	*	***
Sakamoto	****	**	***
Suda	****	**	***
Wu	****	**	***

**Table 4:** Summary of findings table.

Outcomes	№ of participants	Certainty of the evidence (GRADE)	Relative effect (95% CI)	Anticipated absolute effects	
				Risk with no-OAC	Risk difference with OAC
Any stroke/SE or cardiac death	3,021	⨁⨁⨁◯ Moderate^a^	OR 0.66 (0.33 to 1.29)	237 per 1 000	67 fewer per 1,000 (144 fewer to 49 more)
Major cardiovascular ischemic events	918	⨁⨁⨁⨁ High^a^	OR 0.11 (0.03 to 0.45)	332 per 1 000	280 fewer per 1,000 (317 fewer to 149 fewer)
Major bleeding	4,296	⨁⨁⨁◯ Moderate^a^	OR 0.63 (0.23 to 1.72)	80 per 1 000	28 fewer per 1,000 (61 fewer to 50 more)
Mortality from any cause	7,515	⨁⨁⨁◯ Moderate^a^	OR 0.75 (0.50 to 1.11)	222 per 1 000	46 fewer per 1,000 (97 fewer to 19 more)
Ischemic stroke	5,032	⨁⨁⨁◯ Moderate^a^	OR 0.50 (0.37 to 0.68)	76 per 1 000	36 fewer per 1,000 (46 fewer to 23 fewer)
ICH recurrence	4,144	⨁⨁⨁◯ Moderate^a^	OR 1.25 (0.28 to 5.71)	55 per 1 000	13 more per 1,000 (39 fewer to 194 more)

*The risk in the intervention group (and its 95% confidence interval) is based on the assumed risk in the comparison group and the relative effect of the intervention (and its 95% CI). CI: confidence interval; OR: odds ratio. GRADE Working Group grades of evidence, High certainty: we are very confident that the true effect lies close to that of the estimate of the effect. Moderate certainty: we are moderately confident in the effect estimate: the true effect is likely to be close to the estimate of the effect, but there is a possibility that it is substantially different. Low certainty: our confidence in the effect estimate is limited: the true effect may be substantially different from the estimate of the effect. Very low certainty: we have very little confidence in the effect estimate: the true effect is likely to be substantially different from the estimate of effect.

a Low heterogeneity.

## Discussion

This systematic review and meta-analysis explored the complex balance of risks and benefits associated with the use of DOACs in patients with AF who also have a history of ICH. Our findings indicate that DOACs significantly reduce ischemic stroke risk compared to no therapy or antiplatelet agents without a corresponding increase in mortality or major bleeding events. These results align with prior observational studies suggesting that DOACs may offer a more favorable safety profile than traditional anticoagulants like vitamin K antagonists in this high-risk group. Thus, our findings may support the use of DOACs as a viable strategy for stroke prevention despite prior ICH, especially in those with high stroke risk scores.

Patients with AF are at a heightened risk of IS correlated to the CHA2DS2-VASc score, notably those with a history of stroke or transient ischemic attack being the strongest predictor of future strokes (7, 38). Consequently, preventing recurrent strokes in individuals with AF and a prior ischemic stroke or TIA is a critical priority in cerebrovascular care. Closing the auricle can be an alternative for these patients (3–5), but in current practice, this is not retained or contraindicated in many cases. Thus, the choice is then limited between resuming Warfarin, DOACs, antiplatelet agents, and therapeutic abstention. In numerous countries, before the availability of DOACs, only 50 to 66% of patients with AF were treated with warfarin (39). Besides, reluctance to prescribe warfarin for patients with AF may sometimes be justified, as intracranial hemorrhage during follow-up is a significant predictor of poor long-term functional outcomes following an ischemic stroke or transient ischemic attack (40). The possibility of another intracranial hemorrhage remains a concern and deciding to resume anticoagulation therapy is a challenging one for physicians. However, a Danish nationwide cohort study showed that Oral anticoagulant therapy has been shown to significantly reduce rates of ischemic stroke and all-cause mortality, suggesting that reintroducing anticoagulants after intracranial hemorrhage is a viable option (32). This finding may support the use of DOACs as a viable strategy for stroke prevention despite prior ICH, especially in those with high stroke risk scores. However, our analysis did not show a significant difference in ICH recurrence or major bleeding rates between the DOAC and non-therapy/antiplatelet groups, suggesting that the anticipated bleeding risk associated with anticoagulation may be less substantial than previously thought for DOACs. A meta-analysis published in 2023 by Al-Shahi Salman et al. (26), assessed the effects of starting versus avoiding DOACs in patients with spontaneous intracranial hemorrhage. The study demonstrated that DOACs lowered the risk of ischemic major adverse cardiovascular events. However, it had several limitations, including the inclusion of only four trials with a total of 412 patients in the final analysis. Furthermore, the researchers were unable to conclude the risk of hemorrhagic major adverse cardiovascular events, mortality, or functional outcomes.

This absolute reduction in the risk of ischemic major adverse cardiovascular events appears to outweigh the potential increase in the risk of hemorrhagic major adverse cardiovascular events. Yet, the overall net benefit of oral anticoagulation has to be fully established. Additionally, hemorrhagic events are more likely to lead to death or disability compared to ischemic events. However, it remains uncertain whether an overall reduction in the absolute risk of major adverse cardiovascular events translates into a net reduction in mortality or long-term dependence.

The main critical limitation of this study is the absence of stratification based on the type and location of intracranial hemorrhage. We were not able to distinguish patients with lobar intracerebral hemorrhage or non-aneurysmal convexity subarachnoid hemorrhage—subgroups associated with a higher risk of recurrence, often due to cerebral amyloid angiopathy—from those with deep hemorrhages across all endpoints. Although our meta-analysis included a subset of 103 participants from these higher-risk groups and found no clear interaction between ICH location and the net effect of oral anticoagulation, our findings contrast with preliminary safety concerns raised in the ENRICH-AF trial. In that trial, enrollment of patients with lobar ICH was halted due to an unacceptable rate of recurrent hemorrhagic stroke in the edoxaban group. These emerging findings, though unpublished, raise uncertainty that underscores the need for ongoing trials to further clarify the role of anticoagulation in this subgroup. Additionally, our analysis is limited by the lack of patient-level data on important confounders such as comorbidities, hypertension control, and prior use of combination antiplatelet-anticoagulant therapy. The inclusion of both randomized controlled trials and controlled clinical trials introduces methodological variability and potential bias in patient selection and outcome reporting. Data availability also varied significantly across outcomes: for example, ischemic stroke was reported in only a portion of the total cohort due to inconsistent outcome reporting across studies. Moreover, definitions of comparator groups differed; some control groups included antiplatelet therapy, and others had no therapy or placebo, which may affect the comparability of outcomes. Although a stratified analysis of OAC vs. antiplatelet and OAC vs. no therapy would be insightful, the available data did not consistently support such distinctions. Finally, follow-up duration was heterogeneous, and long-term data, especially from ongoing trials such as ASPIRE, ENRICH-AF, and STROKECLOSE, are still lacking. While DOACs showed promise in reducing ischemic events, uncertainty remains regarding their safety profile, particularly with respect to bleeding and ICH recurrence, as evidenced by the wide confidence intervals. Future research should prioritize individualized risk stratification, standardized outcome reporting, and longer follow-up to better inform anticoagulation strategies after intracranial hemorrhage.

## Conclusion

To conclude, our findings suggest that DOACs substantially lower the risk of ischemic stroke compared to no treatment or antiplatelet therapy without a notable rise in mortality or major bleeding events. These outcomes are consistent with previous observational studies indicating that DOACs may provide a safer alternative to traditional anticoagulants, such as vitamin K antagonists, for this high-risk population. These findings should serve as motivation to support the recruitment efforts and the successful completion of ongoing clinical trials.

## Data Availability

The raw data supporting the conclusions of this article will be made available by the authors, without undue reservation.
