# Supplementary material for: Direct oral anticoagulation versus no therapy or antiplatelet for stroke prevention in patients with atrial fibrillation and history of intracranial hemorrhage: a systematic review and meta-analysis
Source: Front Med (Lausanne). 2025 Apr 25;12:1570809. doi: 10.3389/fmed.2025.1570809 (PMC12062129; doi:10.3389/fmed.2025.1570809)
Supplement: Supplementary file 1 [file Data_Sheet_1.DOCX]

| The different search strategies | | | | |
| --- | --- | --- | --- | --- |
| PubMed | EmBase | Cochrane | Scopus | Web of Science |
| “atrial fibrillation”[MeSH Terms] AND (“brain hemorrhage”[Title/Abstract] OR “intracerebral hemorrhage”[Title/Abstract] OR “cerebral hemorrhage”[Title/Abstract] OR “intracranial hemorrhage”[Title/Abstract]) AND (“Anticoagulants”[MeSH Terms] OR “warfarin”[Title/Abstract] OR “vitamin k antagonist”[Title/Abstract] OR NOAC”[Title/Abstract] OR “novel oral anticoagulant”[Title/Abstract] OR “non vitamin K anticoagulant”[Title/Abstract] OR “DOAC”[Title/Abstract] OR “rivaroxaban”[Title/Abstract] OR “edoxaban”[Title/Abstract] OR “apixaban”[Title/Abstract] OR “dabigatran”[Title/ Abstract]) AND “randomized controlled trial”[MeSH Terms] | #1. ‘atrial fibrillation’/exp #2. ‘brain hemorrhage’:ab,ti #3. ‘intracranial hemorrhage’:ab,ti #4. ‘intracerebral hemorrhage’:ab,ti #5. ‘cerebral hemorrhage’:ab,ti #6. #2 OR #3 OR #4 OR #5 #7. ‘anticoagulant agent’/exp #8. ‘warfarin’:ab,ti #9. ‘vitamin k antagonist’:ab,ti #10. ‘noac’:ab,ti #11. ‘novel oral anticoagulant’:ab,ti #12. ‘non vitamin k anticoagulant’:ab,ti #13. ‘doac’:ab,ti #14. ‘rivaroxaban’:ab,ti #15. ‘dabigatran’:ab,ti #16. ‘apixaban’:ab,ti #17. ‘edoxaban’:ab,ti #18. ‘randomized controlled trial’/exp  #19. 7 OR #8 OR #9 OR #10 OR #11 OR #12 OR #13 OR #14 OR #15 OR #16 OR #17 #20. #1 AND #6 AND #18 AND #19 | #1 MeSH descriptor: [Randomized controlled trial] explode all trees #2 MeSH descriptor: [Atrial Fibrillation] explode all trees #3 MeSH descriptor: [Intracranial Hemorrhages] explode all trees #4 (“intracerebral hemorrhage”):ti,ab,kw #5 (cerebral hemorrhage):ti,ab,kw #6 #3 or #4 or #5 #7 MeSH descriptor: [Anticoagulants] explode all trees #8 (anticoagulation):ti,ab,kw OR (NOAC):ti,ab,kw OR (novel oral anticoagulant):ti,ab,kw OR (non vitamin k antagonist):ti,ab,kw AND (“Warfarin”):ti,ab,kw (Word variations have been searched) #9 (DOAC):ti,ab,kw OR (rivaroxaban):ti,ab,kw OR (edoxaban):ti,ab,kw OR (apixaban):ti,ab,kw AND (dabigatran):ti,ab,kw (Word variations have been searched) #10 #8 or #9 #11 #1 and #2 and #6 and #10 | (TITLE-ABS-KEY("atrial fibrillation")) AND (TITLE-ABS-KEY("brain hemorrhage" OR "intracerebral hemorrhage" OR "cerebral hemorrhage" OR "intracranial hemorrhage")) AND (TITLE-ABS-KEY("anticoagulants" OR "warfarin" OR "vitamin k antagonist" OR "noac" OR "novel oral anticoagulant" OR "non vitamin K anticoagulant" OR "doac" OR "rivaroxaban" OR "dabigatran" OR "apixaban" OR "edoxaban")) AND (TITLE-ABS-KEY("randomized controlled trial")) | TS=("atrial fibrillation") AND TS=("brain hemorrhage" OR "intracerebral hemorrhage" OR "cerebral hemorrhage" OR "intracranial hemorrhage") AND TS=("anticoagulants" OR "warfarin" OR "vitamin K antagonist" OR "noac" OR "novel oral anticoagulant" OR "non vitamin K anticoagulant" OR "doac" OR "rivaroxaban" OR "dabigatran" OR "apixaban" OR "edoxaban") AND TS=("randomized controlled trial") |
